# Comparison of Chemical Composition and Bioactivities of Essential Oils from Fresh and Dry Rhizomes of *Zingiber zerumbet* (L.) Smith

**DOI:** 10.1155/2020/9641284

**Published:** 2020-02-11

**Authors:** Minyi Tian, Xianghuan Wu, Yi Hong, Huijuan Wang, Guodong Deng, Ying Zhou

**Affiliations:** ^1^Key Laboratory of Plant Resource Conservation and Germplasm Innovation in Mountainous Region (Ministry of Education), Collaborative Innovation Center for Mountain Ecology & Agro-Bioengineering (CICMEAB), College of Life Sciences, Institute of Agro-bioengineering, Guizhou University, Guiyang 550025, China; ^2^College of Pharmacy, Guizhou University of Traditional Chinese Medicine, Guiyang 550025, China

## Abstract

The chemical constituents and the antioxidant, antimicrobial, and cytotoxic activities of fresh rhizome essential oil (FR-EO) and dry rhizome essential oil (DR-EO) of *Zingiber zerumbet* (L.) Smith obtained from Southwest China were compared. Zerumbone was the predominant component in both FR-EO and DR-EO (75.0% and 41.9%, respectively). FR-EO, DR-EO, and zerumbone were all demonstrated to have significant antimicrobial capacity against *Staphylococcus aureus*, *Bacillus subtilis*, *Escherichia coli*, and *Proteus vulgaris*, with minimum inhibitory concentration (MIC) ranging from 31.25 to 156.25 *μ*g/mL and minimum bactericidal concentration (MBC) ranging from 62.50 to 625.00 *μ*g/mL. Zerumbone showed the strongest antimicrobial potential against all tested microorganisms compared with the fresh and dry rhizome essential oils. FR-EO was found to be more active than DR-EO against *Staphylococcus aureus*, *Bacillus subtilis*, *Escherichia coli*, and *Proteus vulgaris*. FR-EO, DR-EO, and zerumbone all showed significant cytotoxic activity against K562, PC-3, and A549 human tumor cell lines in a time- and concentration-dependent manner. Zerumbone exhibited the strongest antiproliferative activity against all tested human tumor cell lines with an IC_50_ of 4.21–11.09 *μ*g/mL for 72 h incubation, as compared with the fresh and dry rhizome oils. The cytotoxic activity of FR-EO (IC_50_: 10.48–14.51 *μ*g/mL for 72 h) was found to be significantly higher (*p* < 0.05) than that of DR-EO (IC_50_: 13.83–33.24 *μ*g/mL for 72 h). FR-EO, DR-EO, and zerumbone exhibited selective cytotoxic activity to tumor cells, with a significantly low cytotoxicity to normal cells (MRC-5, IC_50_: 56.98–147.29 *μ*g/mL). However, FR-EO, DR-EO, and zerumbone all exhibited weak free-radical-scavenging activity according to DPPH and ABTS analysis. The findings highlighted in this study show that FR-EO provides appreciably higher content of the bioactive compound, zerumbone, and has higher antimicrobial and cytotoxic properties than DR-EO. Thus, fresh *Z. zerumbet* rhizome should be preferred in cosmetic, food, and pharmaceutical applications.

## 1. Introduction

The genus *Zingiber* includes about 141 species and is an important source of essential oil that is widely used in the perfume, cosmetic, and pharmaceutical industries [1–3]. *Zingiber zerumbet* (L.) Smith is an aromatic and tuberose plant of this genus and is commonly known as *hong qiu jiang* in China. *Z. zerumbet* has received great interest from scientists for its spice and medicinal values [4, 5]. It is mainly distributed in tropical and subtropical regions including Sri Lanka, Nepal, Bangladesh, Malaysia, India, and Southwest China [6]. It is a food and medicinal plant with great cultivation potential because of its low planting costs [7]. Both fresh and dry rhizomes of *Z. zerumbet* are widely used for spice, beverage, and medicinal purposes [6, 8]. Traditionally, its rhizomes are used to treat cold, cough, fever, stomach cramps, flatulence, colic pain, swelling, loss of appetite, inflammation, leprosy, sore throat, bacterial infections, allergies, and skin diseases [7, 9]. In China, *Z. zerumbet* rhizome is an edible vegetable and is used as a traditional Chinese medicine for the treatment of abdominal pain and diarrhea.

*Z. zerumbet* rhizome oil is used in perfumes and has been studied extensively because of its high medicinal value [10]. The chemical constituents of *Z. zerumbet* rhizome essential oil from India, Malaysia, Indonesia, Bangladesh, Reunion Island, Fiji, Vietnam, Philippines, Thailand, Polynesia Islands, and Japan have been studied, and the results indicate that the chemical constituents of rhizome essential oil varied according to geographical location [4, 9, 11–27]. The *Z. zerumbet* rhizome oil is a mixture of terpenes and contains zerumbone as the major constituent [4, 12–14]. The zerumbone and *Z. zerumbet* rhizome oil have been demonstrated to have a variety of pharmacological activities, including chemopreventive [10], chemotherapeutic [10], antiproliferative [12], antinociceptive [22], antimicrobial [12, 28], antitumor [28, 29], antihypersensitivity [30], antioxidant [30, 31], antisecretory [31], and anti-inflammatory [10, 32, 33] properties. To our knowledge, the chemical constituents and pharmacological activities of the *Z. zerumbet* rhizome volatile oil from Southwest China have not been reported.

Aromatic herbs are sensitive to drying processes, which can lead to the loss of biological activity [34]. In the previous literature, the chemical constituents of *Z. zerumbet* fresh or dried rhizome essential oils were studied; the results indicate that the zerumbone content in the fresh rhizome essential oil from different geographical locations varied from 8.1 to 84.8% [4, 9, 12–24] and the zerumbone content of the dry rhizome oil from different geographical locations varied between 1.2 and 35.5% [25–27]. To our knowledge, the effect of drying on the chemical constituents and pharmacological activities of the volatile oil of *Z. zerumbet* rhizome has not been reported. Hence, this study was designed to compare the chemical composition and antioxidant, antimicrobial, and cytotoxic activities of fresh and dry *Z. zerumbet* rhizome essential oils. At the same time, the zerumbone, as a major bioactive constituent, was purified and compared with the bioactivities of volatile oils from fresh and dry rhizomes.

## 2. Materials and Methods

### 2.1. Plant Material

Fresh *Z. zerumbet* rhizomes were collected from Guangxi Province, China, in September 2017. A portion of the fresh rhizomes was left to dry in a laboratory air-ventilated oven dryer at a temperature of 40°C for 72 h. The identity of the species was confirmed by Prof. Shenghua Wei of Guizhou University of Chinese Medicine. A voucher specimen (no. 1994) was deposited at Guizhou Engineering Center for Innovative Traditional Chinese Medicine and Ethnic Medicine, Guizhou University.

### 2.2. Essential Oils' Extraction

Fresh and dry *Z. zerumbet* rhizomes were cut into pieces and separately placed in a Clevenger-type apparatus and submitted to hydrodistillation (5 h). Essential oils were dried over anhydrous Na_2_SO_4_, filtered, and stored in amber bottles at 4°C.

### 2.3. Chromatographic Analysis

The GC-FID analysis was carried out with an Agilent 6890 gas chromatograph (GC) system coupled to a flame-ionization detector (FID) and equipped with a HP-5MS capillary column (60 m × 0.25 mm × 0.25 *μ*m film thickness). The injection volume was 1 *μ*L (split ratio at 1 : 100) with helium as the carrier gas (flow rate: 1 mL/min). Oven temperature was as follows: 46°C (2 min), 3°C/min to 190°C (48 min), and 15°C/min to 310°C (8 min). The GC-MS analysis was carried out with an Agilent 6890 GC system fitted with an Agilent 5975C MS and a HP-5MS fused silica column. GC parameters were as described above. MS was set to EI mode of 70 eV with mass range (*m*/*z* 29 to 500). The percentage of chemical component was calculated by the peak area normalization method. The experimental retention index was calculated by injecting a series of *n*-alkanes (C_9_–C_18_). The constituents of essential oils were identified by comparison with retention index and mass spectral data in the NIST 14 and Wiley 275 databases.

### 2.4. Isolation and Identification of Zerumbone

Zerumbone was isolated from the essential oil of fresh rhizomes using the recrystallization method. Fresh rhizome essential oil (5 g) was dissolved in hexane (10 mL) and crystallized in a refrigerator at −20°C for 30 min and then filtered. Recrystallization in hexane at −20°C for 30 min and then filtration gave a white crystalline compound. High-performance liquid chromatography (HPLC) was used to confirm the purity of the isolated compound (purity > 99.0%). The white crystals were identified as zerumbone based on a range of detection methods (EI-MS, ^1^H NMR, and ^13^C NMR). EI-MS *m/z* (%): 218 [M+], 203, 189, 175, 163, 150, 135 (100), 121, 107, 96, 79, 67, 53, 41. ^1^H NMR (CDCl_3_, 400 MHz), *δ*: 0.99 (3H, s, H-15), 1.13 (3H, s, H-14), 1.46 (3H, s, H-12), 1.72 (3H, s, H-13), 1.75–1.88 (1H, m, H-1), 2.12–2.30 (4H, m, H-1, H-4, H-5), 2.30–2.43 (1H, m, H-5), 5.18 (1H, bd, *J* = 15.1 Hz, H-2), 5.78 (1H, d, *J* = 16.4 Hz, H-10), 5.89 (1H, d, *J* = 16.4 Hz, H-9), 5.92–5.96 (1H, m, H-6); ^13^C NMR (CDCl_3_, 100 MHz), *δ*: 11.8 (C-13), 15.3 (C-12), 24.3(C-15), 24.5 (C-5), 29.5 (C-14), 37.9 (C-11), 39.5 (C-4), 42.4 (C-1), 125.0 (C-2), 127.2 (C-9), 136.3 (C-3), 137.9 (C-7), 148.9 (C-6), 160.7 (C-10), 204.3 (C-8). The white crystals were identified as zerumbone by comparison with the reported spectral data [4, 35].

### 2.5. Antioxidant Activity

The 1,1-diphenyl-2-picrylhydrazyl (DPPH) free-radical-scavenging capacity of fresh and dry rhizome oils and zerumbone was determined according to the method reported previously [36] with minor modification. Butylated hydroxytoluene (BHT) and ascorbic acid were used as positive controls. Various concentrations of oils and zerumbone solution (1 mL) were mixed with DPPH methanol solution (0.1 mM, 1 mL) and allowed to stand at 37°C for 30 min. The absorbance of the samples was measured with a spectrophotometer at 517 nm.

The 2,2′-azino-bis-3-ethylbenzthiazoline-6-sulphonic acid (ABTS) radical-scavenging capacity of oils and zerumbone was determined according to the method reported by Re et al. [37] with marginal modifications. ABTS solution was generated by reacting 7 mM ABTS solution with 2.45 mM K_2_S_2_O_8_; the mixture was then allowed to stand at 37°C for 16 h. Before experiments, ABTS was diluted with methanol to obtain an ABTS solution with an absorbance of 0.70 ± 0.02 at 734 nm. The ABTS solution (4 mL) was added to various concentrations of sample solution (0.4 mL), and then, the mixture was incubated at 37°C for 10 min in the dark. The results are expressed using the IC_50_ value and ascorbic acid equivalent antioxidant capacity (AEAC) value. All measurements were repeated three times.

### 2.6. Antimicrobial Activity

Antimicrobial tests were performed with seven microbial strains of *Staphylococcus aureus* (ATCC 6538P), *Enterococcus faecalis* (ATCC 29212), *Pseudomonas aeruginosa* (CMCC (B) 10104), *Bacillus subtilis* (CMCC (B) 63501), *Escherichia coli* (ATCC 25922), *Proteus vulgaris* (CMCC (B) 49027), and *Candida albicans* (CMCC (F) 98001). Antimicrobial activities of FR-EO, DR-EO, and zerumbone were evaluated by using the disc agar diffusion assay reported previously [38] with minor modification. FR-EO, DR-EO, and zerumbone solutions were diluted with ethyl acetate (100 mg/mL). Filter paper discs (diameter 6 mm) containing oils and zerumbone solution (20 *μ*L) were incubated at 37°C for 24 h, and the inhibition zone diameter (including the 6 mm disk) was recorded. The MIC and MBC values of FR-EO, DR-EO, and zerumbone were evaluated by the broth microdilution assay described previously [39] with slight modification. The FR-EO, DR-EO, and zerumbone were initially diluted in DMSO and later in Mueller–Hinton broth or Sabouraud dextrose broth. The tested sample solution of twofold dilution (100 *μ*L) was transferred into each well. The inoculum was added to all wells. The final concentration of the bacterial cells in the wells was approximately 5 × 10^5^ CFU/mL. The 96-well plates were incubated for 24 h at 37°C, and then, 10 *μ*L of resazurin aqueous solution (0.01%) was added to the 96-well plates as an indicator of microbial growth by detecting the reduction of blue dye resazurin to pink resorufin. The 96-well plates were incubated at 37°C for 2 h in the dark. The MIC was defined as the lowest concentration of the sample when the resazurin color changed. To obtain the MBC value, 10 *μ*L samples were obtained from the wells (no color change) and subcultured in agar plates, and the MBC was determined as the lowest concentration without any microbial growth after 24 h at 37°C [40]. Each test was repeated in triplicate.

### 2.7. Cytotoxic Activity

Human prostatic carcinoma (PC-3), leukemic (K562), lung cancer (A549), and fetal lung fibroblasts (MRC-5) cell lines were maintained in RPMI 1640 medium (2 mM glutamine, 10% fetal bovine serum, 100 U/mL penicillin, and 100 U/mL streptomycin) and incubated in a humidified incubator at 37°C with 5% CO_2_ atmosphere. The cytotoxic activity was evaluated by MTT assay with slight modification [41]. The cells were seeded at a density of 5 × 10^3^ cells per well in 80 *μ*L of culture medium and incubated for 24 h before treatment. The FR-EO, DR-EO, and zerumbone were dissolved in DMSO and then serially double diluted with the medium. The diluted sample solution (20 *μ*L) was added to each well and incubated for 24, 48, and 72 h. The medium was then removed, and 10 *μ*L of MTT solution (5 mg/mL in PBS) was added to the wells and cultured for 4 h. Formazan crystals in each well were dissolved by the addition of DMSO (150 *μ*L). A microplate spectrophotometer was used to measure and record the optical density at 490 nm. The results of cytotoxic activity were expressed using the IC_50_ value. Each test was repeated in triplicate.

### 2.8. Statistical Analysis

The results of the tests were repeated in triplicate and expressed as the means ± SD. SPSS software (version 19.0) was used for statistical analysis. Data were compared by one-way analysis of variance (ANOVA) using Tukey's multiple range tests, which was significant at *p* < 0.05.

## 3. Results and Discussion

### 3.1. Chemical Composition

**Figure 1:**
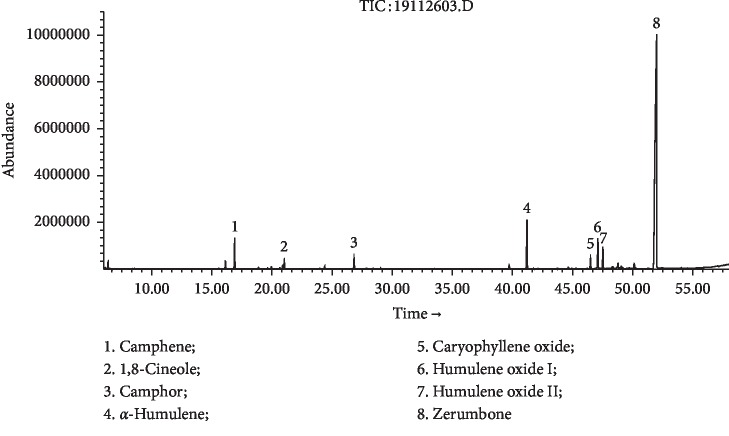
GC-MS chromatogram of *Z. zerumbet* FR-EO.

**Figure 2:**
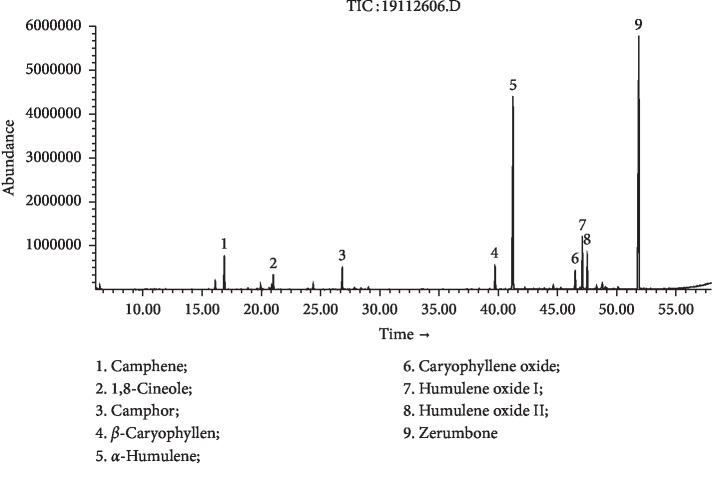
GC-MS chromatogram of *Z. zerumbet* DR-EO.

Hydrodistillation of fresh and dry *Z. zerumbet* rhizomes yielded essential oils in 0.65% and 0.39% (w/w) of fresh weight, respectively. In the present study, crystals were observed during hydrodistillation. Zerumbone crystals were uniformly mixed with rhizome oils and were analyzed by using GC-FID/MS (Table 1). Thirty-six compounds were identified, accounting for 98.9% of FR-EO. The major component was zerumbone (75.0%), followed by *α*-humulene (6.5%), humulene oxide I (3.8%), camphene (3.3%), humulene oxide II (2.7%), camphor (1.3%), caryophyllene oxide (1.3%), and 1,8-cineole (1.2%) (Figure 1). Thirty-six compounds representing 98.8% of DR-EO were identified, containing mainly zerumbone (41.9%), followed by *α*-humulene (29.4%), humulene oxide I (6.0%), humulene oxide II (3.9%), camphene (3.9%), *β*-caryophyllene (2.5%), camphor (2.4%), caryophyllene oxide (2.1%), and 1,8-cineole (1.6%) (Figure 2). The chemical constituents of *Z. zerumbet* rhizome oil from different geographic regions were found to vary [4, 9, 11–27]. To our knowledge, the chemical constituents of the *Z. zerumbet* rhizome volatile oil from Southwest China have not been reported. Most early studies showed that zerumbone was the main constituent. The zerumbone content in FR-EO from different geographical locations varied from 8.1 to 84.8% [4, 9, 12–24]. In DR-EO from different geographical locations, the zerumbone content varied between 1.2 and 35.5% [25–27]. Zerumbone is a highly volatile compound with a unique odor [42]. Our work was undertaken to compare chemical constituents of essential oils of fresh and dry *Z. zerumbet* rhizomes from a single geographical location. The results showed that the content of zerumbone was higher in fresh rhizome oil than in dry rhizome oil. The loss of zerumbone during oven drying processing steps of the rhizome might be attributed to its high volatility. The FR-EO yield (0.65%) of *Z. zerumbet* was higher than the DR-EO yield (0.39%). Oven drying significantly decreased the content of zerumbone and resulted in a low extraction yield of DR-EO. Zerumbone was isolated from fresh rhizome essential oil by recrystallization and identified by spectroscopic analysis. HPLC was used to verify the purity of zerumbone (purity > 99.0%). This method of extracting zerumbone from fresh rhizome essential oil using only recrystallization is a relatively simple and effective method.

### 3.2. Antioxidant Activity

The antioxidant ability of oils and zerumbone was determined based on DPPH and ABTS assays using ascorbic acid and BHT as positive control (Table 2). The IC_50_ values of DPPH free-radical-scavenging capacity were 17416.04 ± 3274.95, 32385.39 ± 5628.23, and 90293.12 ± 3529.38 *μ*g/mL for DR-EO, FR-EO, and zerumbone, respectively, which were much higher than those of BHT (29.25 ± 1.87 *μ*g/mL) and ascorbic acid (1.57 ± 0.23 *μ*g/mL). In the determination of ABTS radical-scavenging capacity, the IC_50_ values of DR-EO (2884.67 ± 232.71 *μ*g/mL), FR-EO (2926.68 ± 104.28 *μ*g/mL), and zerumbone (10840.13 ± 938.35 *μ*g/mL) were much higher than those of BHT (7.47 ± 0.12 *μ*g/mL) and ascorbic acid (2.16 ± 0.43 *μ*g/mL). In the DPPH and ABTS assays, the greater the IC_50_ value, the weaker the ability to scavenge free radicals. Zerumbone exhibited significantly lower radical-scavenging activity than the fresh and dry rhizome essential oils (*p* < 0.05). The DR-EO showed more radical-scavenging activity but was nonsignificant (*p* > 0.05) compared to the FR-EO. The DR-EO, FR-EO, and zerumbone showed weak radical-scavenging capacity according to DPPH and ABTS assays. However, Hemn et al. [30] reported that zerumbone was an effective antioxidant that inhibits free-radical production in the prevention and treatment of atherosclerosis *in vivo*. The DPPH and ABTS assays can be used to assess the ability to scavenge free radicals but are not suitable for assessing lipid peroxidation inhibition efficiency [43]. Different methods of assessment of antioxidant capacity *in vitro* and *in vivo* may result in inconsistent results.

### 3.3. Antimicrobial Activity

The antimicrobial ability of fresh and dry rhizome essential oils and zerumbone was qualitatively determined based on the disc agar diffusion assay and quantitatively assessed by the broth microdilution assay. The results are presented in Table 3 and Table 4. The fresh and dry rhizome essential oils and zerumbone showed varying degrees of antimicrobial potential. Zerumbone was the most effective, as compared with FR-EO and DR-EO, against all tested strains; the diameter of inhibition zones ranged from 9.48 to 15.72 mm and showed minimal MIC (31.25–250.00 *μ*g/mL) and MBC (62.50–250.00 *μ*g/mL). The MIC and MBC values of FR-EO were the same as those of DR-EO against *E. faecalis*, *P. aeruginosa*, and *C. albicans*, but FR-EO exhibited higher activity than DR-EO against *S. aureus*, *B. subtilis*, *E. coli*, and *P. vulgaris*, with the diameter of inhibition zones (9.36–14.54 mm), MIC (78.13–156.25 *μ*g/mL), and MBC (156.25–312.25 *μ*g/mL). The diameter of inhibition zones, MIC, and MBC for DR-EO against all tested strains ranged from 8.23 to 12.31 mm, 156.25 to 1250.00 *μ*g/mL, and 156.25 to 2500.00 *μ*g/mL, respectively. Zerumbone was reported to exhibit significant to moderate antimicrobial activity and was discovered to be an important antimicrobial active compound [10, 44, 45]. Hence, the antimicrobial capacity of *Z. zerumbet* rhizome oil might be associated with the content of zerumbone. Oven drying significantly reduced the content of zerumbone, which might be responsible for the decrease in the antimicrobial ability of *Z. zerumbet* rhizome essential oil.

### 3.4. Cytotoxic Activity

The antiproliferation activity of fresh and dry rhizome oils and zerumbone was evaluated against K562, PC-3, and A549 human tumor cell lines and MRC-5 normal human cell line by MTT assay (Table 5). FR-EO, DR-EO, and zerumbone exhibited significant cytotoxicity against all tested tumor cell lines in a time- and concentration-dependent manner. The cytotoxic capacity against tumor cell lines of the oils and zerumbone were, from strongest to weakest: zerumbone > FR-EO > DR-EO, with statistical significance at *p* < 0.05. The zerumbone reduced the proliferation of A549 (IC_50_ = 11.09 ± 0.39 *μ*g/mL), PC-3 (IC_50_ = 7.66 ± 0.68 *μ*g/mL), and K562 (IC_50_ = 4.21 ± 0.84 *μ*g/mL) cell lines after 72 h incubation and showed more cytotoxicity against K562 compared to the positive control (cisplatin). The FR-EO affected the proliferation of A549, PC-3, and K562 cell lines for 72 h incubation with IC_50_ values (10.48–14.51 *μ*g/mL) and was more effective than DR-EO, which had IC_50_ values in the range 13.83 to 33.24 *μ*g/mL for 72 h incubation (*p* < 0.05). The IC_50_ values of FR-EO, DR-EO, and zerumbone against K562, PC-3, and A549 tumor cells were significantly lower compared to those against the normal cell line (MRC-5, IC_50_: 56.98–147.29 *μ*g/mL for 72 h), with statistical significance at *p* < 0.05. Recent studies have shown that zerumbone possessed significant cytotoxic activity against lung cancer [12], colon cancer [46], leukemia [47], ovarian cancer [48], skin cancer [49], liver cancer [50, 51], and breast cancer [52]. Zerumbone from *Z. zerumbet* has been found to be a highly active anticancer natural product [10]. In previous studies, zerumbone had no considerable cytotoxic effect on normal mammalian cells (Vero) up to 100 *μ*g/mL after 48 h incubation [53]. Zerumbone exhibited selective cytotoxic activity to human tumor cell lines, with a significantly lower cytotoxicity to human normal cell line than to tumor cells. The second main compound in both FR-EO and DR-EO was *α*-humulene (10.8% and 28.5%, respectively). The *α*-humulene, a structural analogue lacking only the carbonyl group in zerumbone, did not exhibit anticancer ability [54, 55]. Therefore, drying process negatively affected the cytotoxic capacity of rhizome essential oil suggesting that zerumbone, mostly lost during the process, was mainly responsible of cytotoxic activity.

## 4. Conclusion

To our knowledge, this is the first comparison of the chemical composition and antioxidant, antimicrobial, and cytotoxic activities of *Z. zerumbet* fresh and dry rhizome essential oils. Thirty-six compounds of FR-EO and DR-EO were identified by using GC-FID/MS, and the major component was zerumbone (75.0% and 41.9%, respectively). In addition to their low antioxidant activity, FR-EO, DR-EO, and zerumbone demonstrated significant antimicrobial capacity against *S. aureus*, *B. subtilis*, *E. coli*, and *P. vulgaris* and significant cytotoxic activity against A549, K562, and PC-3 cell lines. Oven drying had a significant effect on the chemical constituents and on the antimicrobial and cytotoxic capacity of the rhizome oil. From GC-MS data, it was clear that the content of zerumbone in DR-EO was considerably less than that in FR-EO. The results of antimicrobial and cytotoxic analysis clearly showed that FR-EO was more effective than DR-EO. Oven drying significantly reduced the content of zerumbone, which was responsible for the decline in antibacterial and cytotoxic properties of rhizome. The fresh *Z. zerumbet* rhizome was found to have more bioactive compound, zerumbone, and showed stronger antimicrobial and cytotoxic properties than the dry rhizome. Thus, fresh *Z. zerumbet* rhizomes should be preferred in cosmetics, food, and pharmaceutical applications.

## Figures and Tables

**Table 1:** Chemical composition of *Z. zerumbet* FR-EO and DR-EO.

Compound^a^	RI^b^	RI^c^	% area		Identification^d^
			DR-EO	FR-EO	
Tricyclene	926	925	0.1	0.1	MS, RI
*α*-Pinene	937	937	1.0	0.8	MS, RI
Camphene	952	952	3.9	3.3	MS, RI
Sabinene	976	974	tr	tr	MS, RI
*β*-Pinene	980	979	tr	tr	MS, RI
*β*-Myrcene	992	991	0.1	0.1	MS, RI
*α*-Phellandrene	1007	1005	0.1	0.1	MS, RI
*δ*-3-Carene	1013	1011	0.5	0.2	MS, RI
*p*-Cymene	1027	1023	0.1	0.1	MS, RI
1,8-Cineole	1034	1032	1.6	1.2	MS, RI
*γ*-Terpinene	1062	1060	tr	tr	MS, RI
Fenchone	1092	1096	0.1	0.1	MS, RI
Linalool	1101	1099	0.6	0.3	MS, RI
Camphor	1149	1145	2.4	1.3	MS, RI
l-Borneol	1170	1167	0.2	0.1	MS, RI
4-Terpineol	1180	1182	0.1	0.1	MS, RI
*α*-Terpineol	1193	1189	0.2	0.1	MS, RI
Verbenone	1212	1204	tr	tr	MS, RI
l-Bornyl acetate	1291	1284	0.1	tr	MS, RI
Isobornyl acetate	1294	1286	0.1	tr	MS, RI
Myrtenyl acetate	1329	1327	tr	tr	MS, RI
*α*-Copaene	1378	1376	tr	tr	MS, RI
*β*-Elemene	1394	1391	0.1	tr	MS, RI
*α*-Gurjunene	1417	1409	0.1	tr	MS, RI
*β*-Caryophyllen	1429	1419	2.5	0.4	MS, RI
*α*-Bergamotene	1440	1435	tr	tr	MS, RI
*α*-Humulene	1467	1454	29.4	6.5	MS, RI
*δ*-Cadinene	1534	1524	0.1	tr	MS, RI
Hedycaryol	1560	1559	0.1	tr	MS, RI
*d*-Nerolidol	1568	1564	0.1	0.1	MS, RI
Caryophyllene oxide	1599	1581	2.1	1.3	MS, RI
Humulene oxide I	1614	1596	6.0	3.8	MS, RI
Humulene oxide II	1625	1606	3.9	2.7	MS, RI
*allo*-Aromadendrene epoxide	1657	1639	1.0	0.8	MS, RI
*β*-Eudesmol	1663	1649	0.3	0.4	MS, RI
Zerumbone	1735	1732	41.9	75.0	MS, RI
Monoterpene hydrocarbons (%)			**5.8**	**4.7**	
Oxygenated monoterpenes (%)			**5.2**	**3.2**	
Sesquiterpene hydrocarbons (%)			**32.2**	**6.9**	
Oxygenated sesquiterpenes (%)			**55.4**	**84.1**	
Total (%)			**98.8**	**98.9**	
Yield (W/W) (%)			**0.39**	**0.65**	

^a^Compounds are listed in order of their elution from a HP-5MS column. ^b^Retention index on the HP-5MS column, calculated using homologous series of C_9_–C_18_ alkanes. ^c^Retention index from NIST 14 and Wiley 275 mass spectral databases. ^d^Identification: MS, based on comparison with Wiley 275 and NIST 14 MS databases; RI, based on comparison of calculated RI with those reported in Wiley 275 and NIST 14 databases. tr: trace (trace < 0.1%).

**Table 2:** Antioxidant ability of FR-EO and DR-EO of *Z. zerumbet* and zerumbone.

Sample	DPPH		ABTS	
	IC_50_ (*μ*g/mL)^a^	AEAC (mg/100 g)^b^	IC_50_ (*μ*g/mL)^a^	AEAC (mg/100 g)^b^
DR-EO	17416.04 ± 3274.95	9.01	2884.67 ± 232.71	74.90
FR-EO	32385.39 ± 5628.23	4.85	2926.68 ± 104.28	73.82
Zerumbone	90293.12 ± 3529.38	1.74	10840.13 ± 938.35	19.93
BHT^c^	29.25 ± 1.87		7.47 ± 0.12	
Ascorbic acid^c^	1.57 ± 0.23		2.16 ± 0.43	

^a^IC_50_: the concentration of sample that affords a 50% reduction in the assay, expressed as the means ± SD of triplicate experiments. ^b^AEAC (ascorbic acid equivalent antioxidant capacity) = (IC_50(AA)_/IC_50(Sample)_) × 10^5^. ^c^BHT and ascorbic acid as positive control.

**Table 3:** Diameter of the inhibition zones of *Z. zerumbet* FR-EO, DR-EO, and zerumbone using the agar disc diffusion method.

Microorganisms	Diameter of the inhibition zones (mm)^a^			
	FR-EO	DR-EO	Zerumbone	Streptomycin
Gram positive				
*Enterococcus faecalis* ATCC 29212	10.41 ± 2.31	9.31 ± 0.69	10.00 ± 0.68	9.74 ± 0.96
*Staphylococcus aureus* ATCC 6538P	14.54 ± 3.78	12.31 ± 1.22	15.72 ± 2.42	19.69 ± 1.63
*Bacillus subtilis* CMCC (B) 63501	9.99 ± 1.86	8.55 ± 1.89	9.48 ± 1.96	9.38 ± 1.21
Gram negative				
*Pseudomonas aeruginosa* CMCC (B) 10104	8.34 ± 0.70	8.23 ± 1.17	9.71 ± 0.97	8.36 ± 1.08
*Escherichia coli* ATCC 25922	9.36 ± 1.98	9.79 ± 1.68	10.85 ± 0.83	11.37 ± 0.88
*Proteus vulgaris* CMCC (B) 49027	11.06 ± 1.01	10.29 ± 1.03	11.71 ± 1.55	17.13 ± 2.14
Fungus				
*Candida albicans* CMCC (F) 98001	10.93 ± 1.13	9.31 ± 1.80	11.31 ± 0.83	Na

^a^The diameter of the inhibition zones (mm) were measured including the diameter of the disk (6 mm). The sample solution: FR-EO, DR-EO, and zerumbone were diluted with ethyl acetate, at a concentration of 100 mg/mL (tested volume: 20 *μ*L); positive control: streptomycin (tested volume: 20 *μ*L, 100 *μ*g/mL).

**Table 4:** MIC and MBC values of *Z. zerumbet* FR-EO, DR-EO, and zerumbone using microdilution assay.

Microorganism	MIC and MBC (*μ*g/mL)^a^							
	FR-EO		DR-EO		Zerumbone		Streptomycin	
	MIC	MBC	MIC	MBC	MIC	MBC	MIC	MBC
Gram positive								
*E. faecalis* ATCC 29212	1250.00	1250.00	1250.00	1250.00	250.00	250.00	0.20	0.78
*S. aureus* ATCC 6538P	78.13	156.25	156.25	156.25	31.25	62.50	0.78	0.78
*B. subtilis* CMCC (B) 63501	78.13	156.25	156.25	312.50	62.50	125.00	0.10	0.20
Gram negative								
*P. aeruginosa* CMCC (B)10104	312.50	625.00	312.50	625.00	250.00	250.00	3.13	12.50
*E. coli* ATCC 25922	156.25	312.25	156.25	625.00	62.50	62.50	3.13	6.25
*P. vulgaris* CMCC (B) 49027	78.13	156.25	156.25	312.50	62.50	62.50	0.20	0.39
Fungus								
*C. albicans* CMCC (F) 98001	312.50	2500.00	312.50	2500.00	31.25	250.00	Na	Na

^a^MIC: minimal inhibitory concentration; MBC: minimal bactericidal concentration; streptomycin as positive control.

**Table 5:** Cytotoxic properties of FR-EO and DR-EO of *Z. zerumbet* and zerumbone.

Sample	Incubation time (h)	IC_50_ (*μ*g/mL)^a^			
		A549^b^	PC-3^c^	K562^d^	MRC-5^e^
FR-EO	24	44.88 ± 1.21	53.32 ± 1.34	35.73 ± 1.72	159.47 ± 9.34
	48	38.64 ± 1.03	21.45 ± 1.18	13.73 ± 0.54	133.82 ± 5.97
	72	14.51 ± 0.61	11.23 ± 0.53	10.48 ± 0.95	106.21 ± 7.34

DR-EO	24	68.06 ± 1.09	77.45 ± 0.46	41.79 ± 1.18	216.99 ± 8.27
	48	56.14 ± 1.76	43.90 ± 1.65	17.22 ± 0.51	164.68 ± 2.71
	72	33.24 ± 0.53	13.83 ± 0.59	14.96 ± 1.18	147.29 ± 4.30

Zerumbone	24	22.40 ± 1.80	30.78 ± 1.31	10.08 ± 0.61	117.96 ± 5.67
	48	19.00 ± 0.72	14.30 ± 1.84	6.24 ± 1.05	79.79 ± 3.17
	72	11.09 ± 0.39	7.66 ± 0.68	4.21 ± 0.84	56.98 ± 1.82

Cisplatin^f^	24	5.17 ± 0.44	40.64 ± 1.38	20.36 ± 0.62	14.58 ± 0.91
	48	3.38 ± 0.61	7.64 ± 0.39	11.53 ± 0.91	5.29 ± 0.67
	72	1.91 ± 0.87	2.16 ± 0.67	5.10 ± 0.54	2.99 ± 0.49

^a^IC_50_: the sample concentration reduced cells growth by 50% (after 24-, 48-, and 72-hour incubation), expressed as the mean ± SD of triplicate experiments. ^b^Human lung cancer cell line. ^c^Human prostatic carcinoma cell line. ^d^Human leukemic cell line. ^e^Human fetal lung fibroblasts cell line. ^f^Cisplatin as positive control.

## Data Availability

The data used to support the findings of this study are available from the corresponding author upon request.

## Supplementary Materials

Supplementary Materials Figure S1: pure zerumbone crystals. Figure S2: mass spectrum spectral data of zerumbone. Figure S3: ^1^H NMR spectral data of zerumbone. Figure S4: ^13^C NMR spectral data of zerumbone. Figure S5: the purity test of zerumbone using an HPLC system.
