# Supplementary material for: Comparison of Chemical Composition and Bioactivities of Essential Oils from Fresh and Dry Rhizomes of Zingiber zerumbet (L.) Smith
Source: Biomed Res Int. 2020 Feb 11;2020:9641284. doi: 10.1155/2020/9641284 (PMC7036102; doi:10.1155/2020/9641284)
Supplement: Supplementary Materials — Figure S1: pure zerumbone crystals. Figure S2: mass spectrum spectral data of zerumbone. Figure S3: 1H NMR spectral data of zerumbone. Figure S4: 13C NMR spectral data of zerumbone. Figure S5: the purity test of zerumbone using an HPLC system. [file 9641284.f1.pdf]

Manuscript ID: 9641284

Manuscript Title: Comparison of Chemical Composition and Bioactivities of Essential Oils from Fresh and Dry Rhizomes of *Zingiber zerumbet* (L.) Smith.

SUPPLEMENTARY FILE:

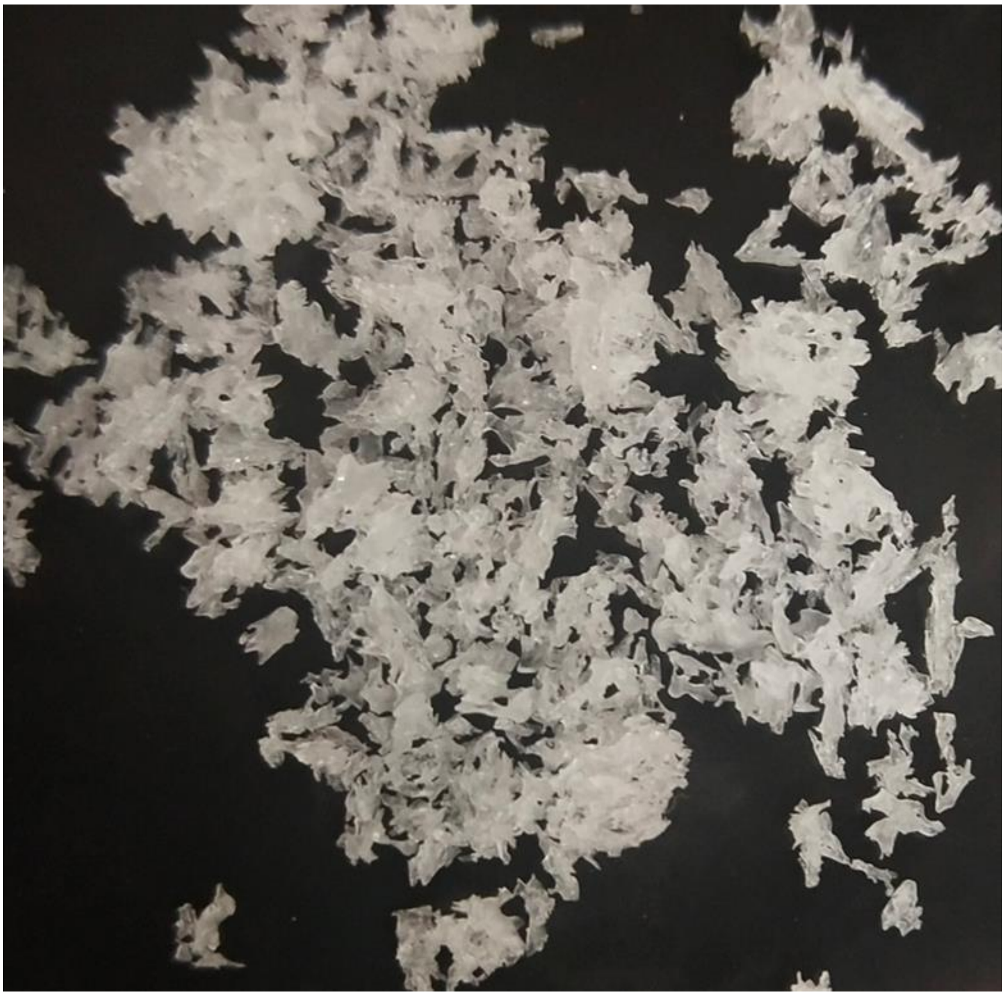

Figure S1. Pure zerumbone crystals

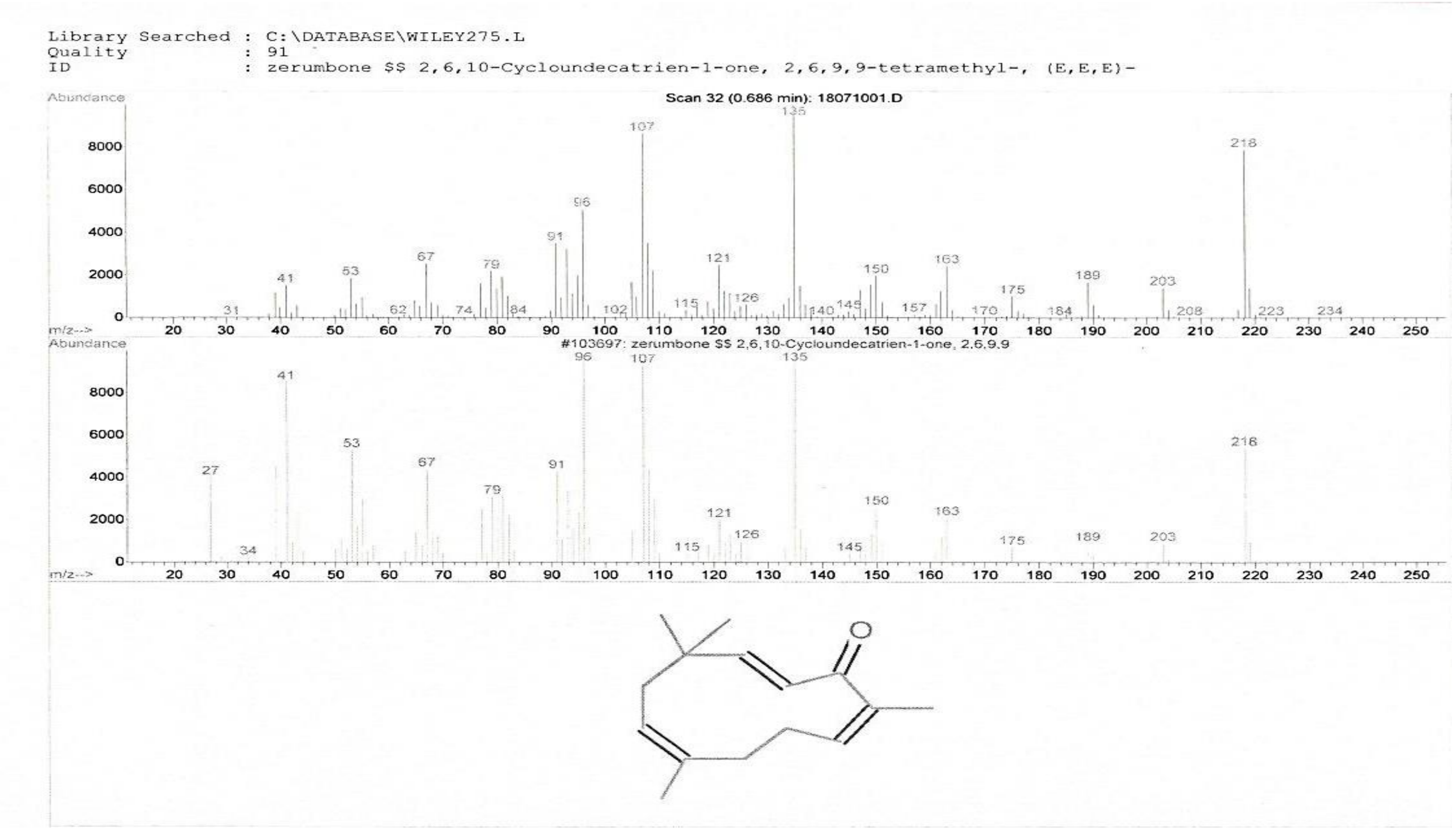

Figure S2. Mass spectrum spectral data of zerumbone

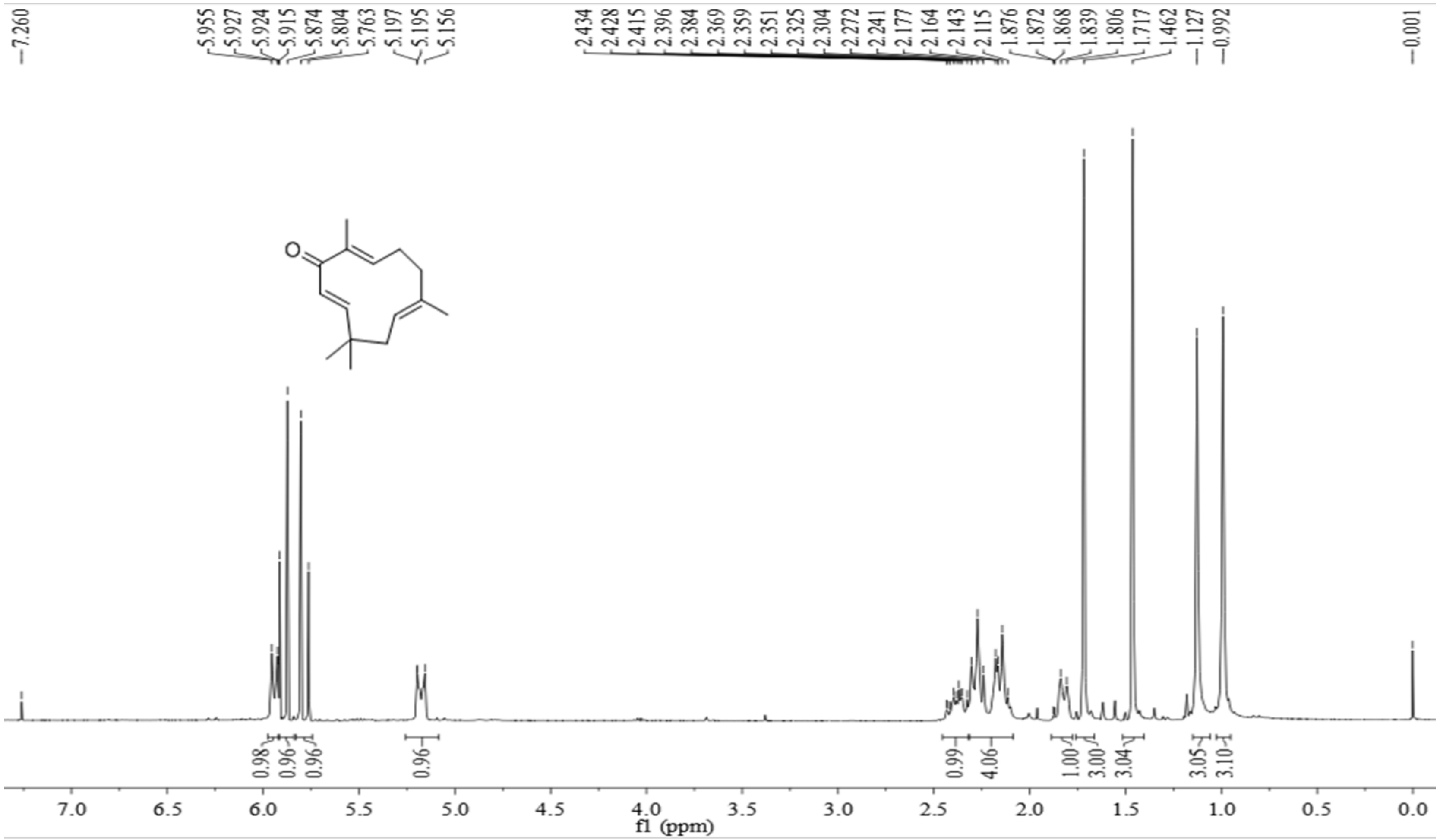

Figure S3. ^{1}H-NMR spectral data of zerumbone

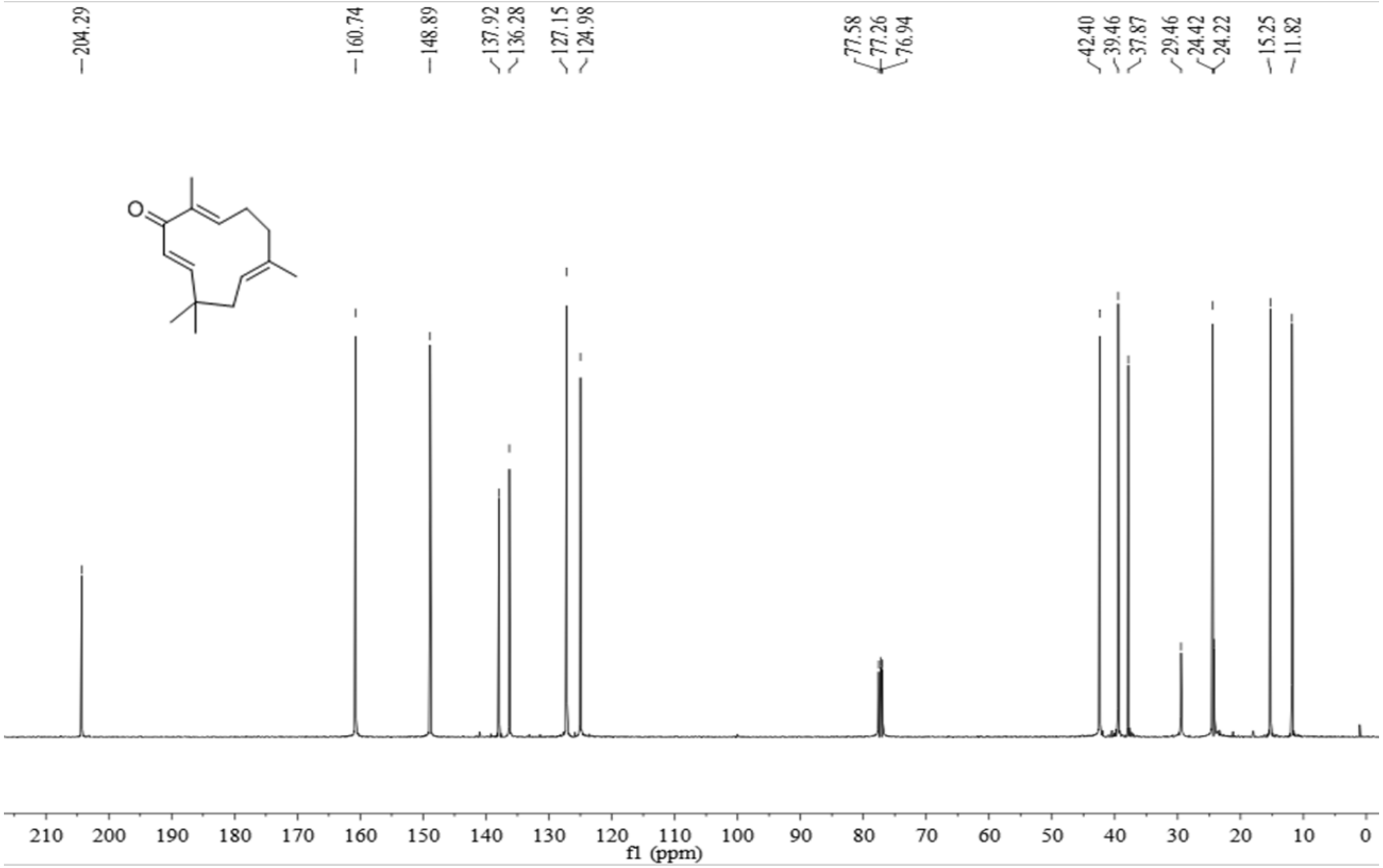

Figure S4. ^{13}C-NMR spectral data of zerumbone

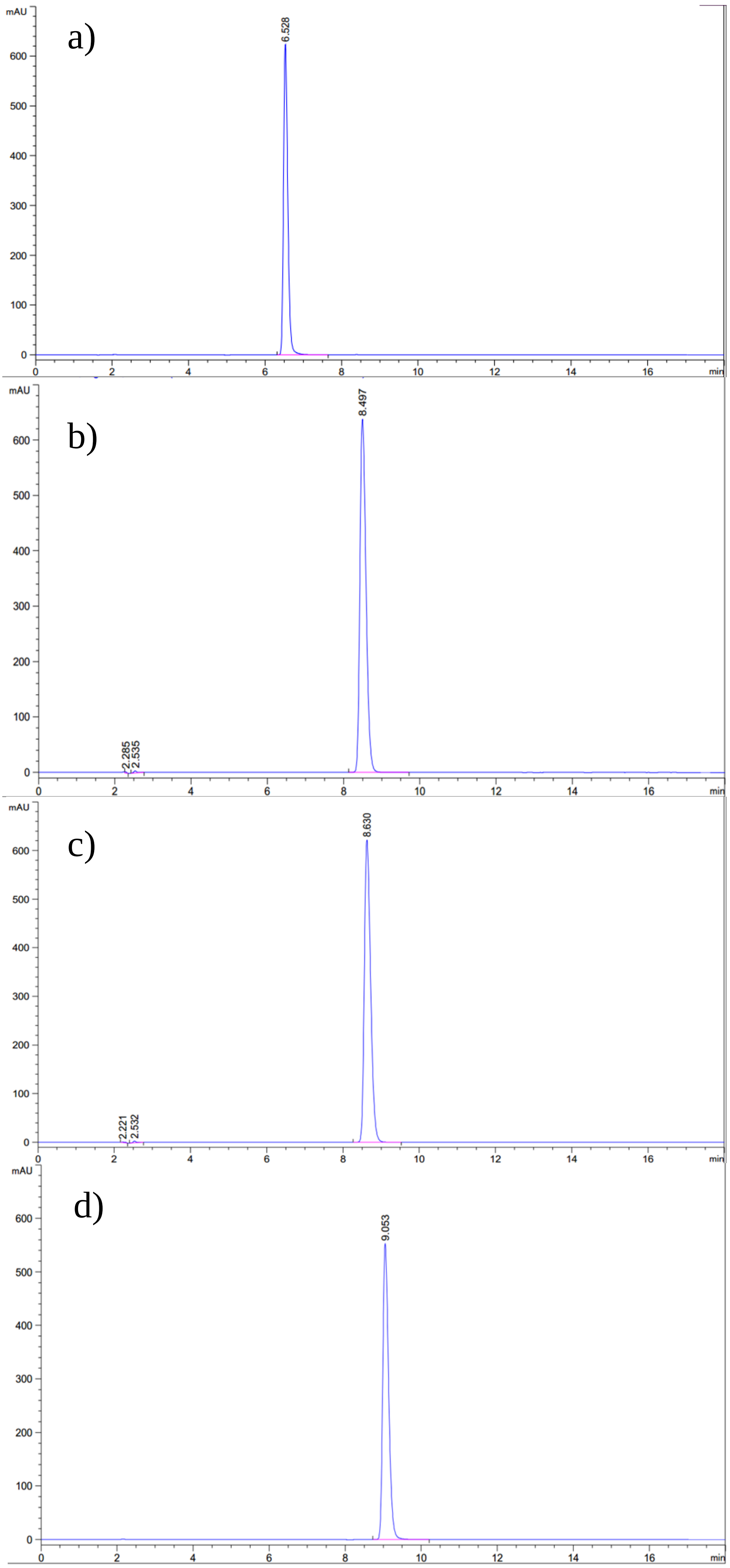

5

Figure S5. The purity test of zerumbone using an HPLC system (Agilent 1260 Infinity, Agilent Technologies, Santa Clara, California, USA) equipped with reversed phase Zorbax SB-C18 column (4.6×250 mm, 5 μm). The column temperature was maintained at 48 ℃, and the injection volume was 10 μL. They were recorded at a wavelength of 280 nm.

a) The isocratic mobile phase was composed of acetonitrile: water (80: 20).

b) The isocratic mobile phase was composed of methanol: 0.01 M formic acid (80: 20).

c) The isocratic mobile phase was composed of methanol: water (80: 20).

d) The isocratic mobile phase was composed of acetonitrile: methanol: water (60: 15: 25).
